# Production of Curcumin-Loaded Silk Fibroin Nanoparticles for Cancer Therapy

**DOI:** 10.3390/nano8020126

**Published:** 2018-02-24

**Authors:** Mercedes G. Montalbán, Jeannine M. Coburn, A. Abel Lozano-Pérez, José L. Cenis, Gloria Víllora, David L. Kaplan

**Affiliations:** 1 Department of Chemical Engineering, Faculty of Chemistry, Regional Campus of International Excellence “Campus Mare Nostrum”, University of Murcia, 30071 Murcia, Spain; gvillora@um.es; 2 Department of Biomedical Engineering, Tufts University, Medford, MA 02155, USA; jeannine.coburn@gmail.com (J.M.C.); david.kaplan@tufts.edu (D.L.K.); 3 Department of Biomedical Engineering, Worcester Polytechnic Institute, Worcester, MA 01609, USA; 4 Department of Biotechnology, Instituto Murciano de Investigación y Desarrollo Agrario y Alimentario (IMIDA), La Alberca, 30150 Murcia, Spain; abel@um.es (A.A.L.-P.); josel.cenis@carm.es (J.L.C.)

**Keywords:** antitumor activity, curcumin, hepatocarcinoma, nanoparticle, neuroblastoma, silk fibroin

## Abstract

Curcumin, extracted from the rhizome of *Curcuma longa*, has been widely used in medicine for centuries due to its anti-inflammatory, anti-cancer, anti-oxidant and anti-microbial effects. However, its bioavailability during treatments is poor because of its low solubility in water, slow dissolution rate and rapid intestinal metabolism. For these reasons, improving the therapeutic efficiency of curcumin using nanocarriers (e.g., biopolymer nanoparticles) has been a research focus, to foster delivery of the curcumin inside cells due to their small size and large surface area. Silk fibroin from the *Bombyx mori* silkworm is a biopolymer characterized by its biocompatibility, biodegradability, amphiphilic chemistry, and excellent mechanical properties in various material formats. These features make silk fibroin nanoparticles useful vehicles for delivering therapeutic drugs, such as curcumin. Curcumin-loaded silk fibroin nanoparticles were synthesized using two procedures (physical adsorption and coprecipitation) more scalable than methods previously described using ionic liquids. The results showed that nanoparticle formulations were 155 to 170 nm in diameter with a zeta potential of approximately −45 mV. The curcumin-loaded silk fibroin nanoparticles obtained by both processing methods were cytotoxic to carcinogenic cells, while not decreasing viability of healthy cells. In the case of tumor cells, curcumin-loaded silk fibroin nanoparticles presented higher efficacy in cytotoxicity against neuroblastoma cells than hepatocarcinoma cells. In conclusion, curcumin-loaded silk fibroin nanoparticles constitute a biodegradable and biocompatible delivery system with the potential to treat tumors by local, long-term sustained drug delivery.

## 1. Introduction

The yellow-orange compound 1,7-bis(4-hydroxy-3-methoxyphenyl)-1,6-heptadiene-3,5-dione (Figure S1), popularly known as curcumin, is the main phenolic pigment extracted from turmeric, the powdered rhizome of *Curcuma longa*, which comprises, approximately, 2–5% of turmeric [1]. Commercial curcumin is a mixture of curcuminoids, containing approximately 77% curcumin, 18% demethoxycurcumin and 5% bisdemethoxycurcumin [2]. This perennial rhizome is commonly cultivated in the Asian continent, especially in India and China. Apart from being used as a spice and flavoring and coloring agent in cooking, curcumin has also been widely employed in Ayurvedic medicine for centuries [3]. The most relevant pharmacological effects of curcumin are its anti-inflammatory [4,5,6,7,8], anti-cancer [2,7,8,9,10,11,12,13], anti-oxidant [8,12,14,15,16] and anti-microbial [7,12,17,18] activities.

Although curcumin possesses remarkable medicinal benefits and has been safe, non-toxic and well-tolerated in animal and human studies, it cannot be administered to patients directly due to its poor solubility in water [1,16,19] (estimated value: 3.12 mg/L at 25 °C [20]). For this reason, the bioavailability of curcumin is limited due to of its reduced absorption. To improve its therapeutic efficiency, a great deal of research has been directed towards improvements in bioavailability. The literature describes several nanocarriers which improve the intra-cellular delivery of curcumin, including solid lipid nanoparticles [21,22,23,24], natural [25,26,27,28,29,30] or synthetic [31,32,33,34,35,36,37,38] polymer-nanoparticles, and inorganic nanoparticles [39,40]. The main advantages of these nanoplatforms are their small size and large surface area, which allows the transport of the nanoparticles through the cell membrane [19,41]. Recently, research interests focus on the use of biopolymers, which are regarded as biodegradable, natural and environmentally friendly, to encapsulate curcumin and other similar drugs [27,42].

Silk fibroin (SF), from the *Bombyx mori* silkworm, is a natural polymeric biomaterial whose main features are its amphiphilic chemistry, biocompatibility, biodegradability, excellent mechanical properties in various material formats, and processing flexibility. All of these properties make SF a useful candidate for sustained and controlled drug release [43]. Several curcumin-loaded SF systems, such as hydrogels, scaffolds and microspheres, have been reported. For example, Li et al. [44] used SF hydrogel films to entrap curcumin and assessed its effect on human bone marrow-derived mesenchymal stem cells related to adipogenic differentiation. Lian et al. [45] incorporated curcumin into copolymeric SF-poly(l-lactic acid-*co*-e-caprolactone) nanofibrous scaffolds, which were evaluated as potential candidates for wound dressings and in tissue engineering. In the same way, Li et al. [46] synthesized copolymeric SF-poly (vinyl alcohol) scaffolds to study the release of curcumin. Kasoju and Bora [47] carried out a similar study but in this case the curcumin-releasing scaffolds were prepared only with SF. Finally, Ratanavaraporn et al. [48] developed gelatin-SF microspheres to study the controlled dual delivery of curcumin and piperine, finding that the curcumin bioavailability increased. However, several reports suggest that SF nanoparticles (SFNs) are more appropriate delivery systems than other SF structures [43,49,50], mainly because of their well-known features of biocompatibility, controlled degradation, size, shape and drug loading and release capacities. By virtue of their small size, SFNs can penetrate thin capillaries, fostering the uptake of drugs by cells. In addition, these SFNs are potential targeted delivery systems because, for instance, they can deliver antitumor drugs to tumor cells. Regarding this application, several pharmaceutical compounds, such as insulin [51], resveratrol [52], cisplatin [53], doxorubicin [54,55,56], paclitaxel [57], indomethacin [58] and quercetin [59], have been loaded into SFNs for delivery. Furthermore, several research groups have studied curcumin encapsulation in SFNs by different techniques [60,61,62,63]. For example, Gupta et al. synthesized curcumin-loaded SFNs (Curc-SFNs) with a size lower than 100 nm using a capillary-microdot technique which is a difficult method in processing and with a low yield [60]. Xie et al. obtained Curc-SFNs with similar size (<100 nm) using solution-enhanced dispersion by supercritical CO_2_ [61]. However, the processing of this method is complicated, and the cost is high. Li et al. fabricated SFNs loaded with curcumin and 5-fluorouracil by self-assembly obtaining sizes higher than 200 nm [62]. Song et al. have synthesized Curc-SFNs with diameter values higher than 100 nm from conventional aqueous SF solutions [63]. 

Recently, Crivelli et al. [64] have reviewed the SFNs preparation methods and highlighted that SFNs can even act as bioactive natural carriers, since they show not only optimal features as inert excipients, but also remarkable intrinsic biological activities such as anti-inflammatory properties. This point is especially interesting because SFNs can be considered bioactive compounds able to improve and support some active principle ingredient effects.

In this work, we studied the synthesis of curcumin-loaded SFNs (Curc-SFNs) to improve on current methods, using ionic liquids to dissolve the SF. Until recently, SFNs have been synthesized by dissolving SF in traditionally used solvents: ionic aqueous solutions, such as 9.3 M LiBr or a 50% (*w*/*v*) CaCl_2_ solution, and ionic hydro-alcoholic solutions such as a CaCl_2_/Ethanol/Water mixture (Ajisawa’s reagent) [65]. These dissolution processes involve exhaustive dialysis in ultrapure water and the subsequent concentration of the SF solution obtained in the process, which is time-consuming and requires large amounts of water. Furthermore, the SF solutions obtained in these processes tend to be unstable and turn into a gel in a relatively short period of time (approximately one week). Apart from using ionic liquids as solvents, we also improved SF dissolution using high-power ultrasound, which requires lower temperature and represents a more efficient energy source compared with thermal energy. While the classical heating method needs hours to complete the dissolution of the SF fiber, the application of ultrasounds to the mixture achieves a significant reduction in the time necessary to complete the SF dissolution process in ionic liquids at less than 100 °C. Therefore, in this work, the solvent effect of the cations and anions of the ionic liquids that break the β-sheet hydrogen bonds network was enhanced by ultrasounds. In addition, ionic liquids can be recycled and used without the loss of their solvent properties in at least four successive cycles. Therefore, using ionic liquids and high-power ultrasounds to dissolve SF constitutes a more environmentally friendly option because the process needs lower temperature, is faster, the SF solutions obtained are very stable and the ionic liquid can be reused in subsequent dissolution processes [66]. All of these reasons, make the synthesis of Curc-SFNs developed in this paper a more scalable and continuous processing option than those already published in the literature.

The main aim of this work was to synthesize Curc-SFNs using a simpler and greener process of SF dissolution and loading the drug through two different procedures (physical adsorption and coprecipitation). Physical-chemical characterization and in vitro cytotoxicity studies were assessed. 

## 2. Materials and Methods 

### 2.1. Materials

*B. mori* silk cocoons were reared in the sericulture facilities of the IMIDA (Murcia, Spain) and raised on a diet of natural *Morus alba* L. fresh leaves. To obtain SF, raw silk cocoons were boiled twice in a 0.05 M Na_2_CO_3_ aqueous solution for 45 min. The remaining SF was rinsed thoroughly with ultrapure water and dried prior to use. SF was dissolved in the ionic liquid 1-ethyl-3-methylimidazolium acetate, [emim^+^][CH_3_COO^−^], by high-power ultrasound, as previously reported [66]. The ionic liquid (95% purity) was purchased from IoliTec GmbH (Frankfurt, Germany) and was used without further purification. Curcumin (99% purity) was purchased from ChromaDex (Irvine, CA, USA). Purified water (18.2 MΩ·cm at 25 °C; from a Millipore Direct-Q1 ultrapure water system, Billerica, MA, USA) was used throughout. All other chemicals and solvents were of analytical grade and were used without further purification.

### 2.2. UV-Vis Spectrophotometric Estimation of Curcumin 

Spectroscopic analysis was carried out using a UV-Vis HELIOSα spectrophotometer (Thermo Scientific, Waltham, MA, USA) and good linear correlations were obtained between absorbance and concentration in the range 0.5–3.5 μg/mL with a correlation coefficient of 0.9974 in water, and in the range 1.0–7.0 μg/mL with a correlation coefficient of 0.9995 in ethanol. The spectrophotometric detection was determined at an absorption maximum of 421 nm using ethanol or water as solvent.

### 2.3. Preparation of SFNs

The preparation of SFNs was based on the method described previously by Lozano-Pérez et al. [66], with modifications. Briefly, an SF-ionic liquid (SIL) solution (10 wt %) was prepared by adding 0.5 g of SF to 4.5 g of [emim^+^][CH_3_COO^−^]. The mixture was treated with a 3/8″ tapered horn of a Sonifier Branson 450D (Emmerson Ultrasonic Corporation, Danbury, CT, USA), with pulsating ultrasonication steps at 30% amplitude at a controlled temperature below 90 °C until complete dissolution. To this solution freshly prepared, 3 mL of ultrapure water was slowly added to reduce viscosity. The final concentration of the SIL solution after diluting with 3 mL of ultrapure water was 6.66 wt %. After heating to 60 °C, the SIL solution was propelled using a peristaltic pump and then sprayed onto 100 mL of gently stirred methanol at −20 °C by a thermostatically controlled 0.7 mm two-fluid nozzle (from a Mini Spray Dryer B-290, BÜCHI Labortechnik, Flawil, Switzerland, Part No. 044698) which uses compressed N_2_ to disperse the solution into fine droplets. A milky white suspension appeared and the suspension was allowed to reach room temperature while stirring for 2 h. Then, the nanoparticle suspension was transferred to centrifuge vials and centrifuged at 13,400 rpm for 15 min, at 4 °C (Sigma 3-18K Centrifuge with a 19,776 H angle rotor, Osterode, Germany). The supernatant, which is free of nanoparticles, was removed and reserved for subsequent recycling of the ionic liquid. An equal volume of fresh methanol was added to the vial, and the white precipitate was suspended by vigorous stirring in a vortex mixer for 2 min and 5 min of ultrasonication with a Branson 450D sonicator (Emmerson Ultrasonic Corporation, Danbury, CT, USA). The centrifugation step was repeated under the same conditions. The white precipitate was subjected to successive rinses with ultrapure water to remove the remaining methanol and ionic liquid. The particles were lyophilized in an Edwards Modulyo 4K Freeze Dryer (Thermo Scientific, Waltham, MA, USA) for 72 h, at −55 °C and 0.5 mbar to obtain dry particles. The methanolic fractions were mixed before recovery of the ionic liquid by removing of the methanol/water on a BÜCHI RE-111 rotary evaporator (Flawil, Switzerland) at 80 °C and 80 mbar. The ionic liquids were kept in a desiccator until reuse. 

### 2.4. Synthesis of Curc-SFNs

The drug was loaded into the SFNs by two different experimental procedures (physical adsorption, Curc-SFNs 1, and coprecipitation, Curc-SFNs 2) to obtain Curc-SFNs. The average yield of the process after lyophilization was of 95.8% and 90.3% for Curc-SFNs 2 and Curc-SFNs 1, respectively. All the experiments have been carried out at least in triplicate.

#### 2.4.1. Drug Loading by Physical Adsorption

For loading of curcumin by physical adsorption, forty milliliters of a 1 mg/mL solution of curcumin in ethanol was used to resuspend 325 mg of SFNs obtained as described in Section 2.3. The suspension was ultrasonicated for 5 min using 30% amplitude with 15 s ON and 15 s OFF pulses and gently stirred at 30 rpm in a MX-RD-ProAnalog Tube Rotator (Scilogex, Rocky Hill, CT, USA) for 24 h. Then, Curc-SFNs 1 were collected by centrifugation at 13,400 rpm for 15 min. Next, the nanoparticles were washed with water to remove the remaining ethanol. The amount of the loaded drug in the SFNs was determined by an indirect method measuring UV-absorbance of curcumin (421 nm) in the supernatants of the centrifugation (in ethanol and in water) and the initial 1 mg/mL solution. 

Drug loading content (DLC) and entrapment efficiency (EE) of the Curc-SFNs 1 obtained were calculated according to the following expressions:

(1) $$\mathrm{DLC}=\frac{\mathrm{Weight}\;\mathrm{of}\;\mathrm{the}\;\mathrm{drug}\;\mathrm{in}\;\mathrm{nanoparticles}}{\mathrm{Weight}\;\mathrm{of}\;\mathrm{the}\;\mathrm{nanoparticles}}\;\times \;100$$

(2) $$\mathrm{EE}\;=\;\frac{\mathrm{Weight}\;\mathrm{of}\;\mathrm{the}\;\mathrm{drug}\;\mathrm{in}\;\mathrm{nanoparticles}}{\mathrm{Weight}\;\mathrm{of}\;\mathrm{the}\;\mathrm{feeding}\;\mathrm{drugs}}\;\times \;100$$

#### 2.4.2. Drug Loading by Coprecipitation

In the case of coprecipitation, the curcumin was loaded during the nanoparticles synthesis process. Briefly, 25 mg of curcumin was dissolved in 3 mL of 0.1 M NaOH solution and the resulting solution was immediately dissolved in 5 g of SIL solution (10 wt %). The curcumin-SIL solution was heated to 60 °C and sprayed onto 100 mL of ethanol using the same equipment as described in Section 2.3. The orange suspension was stirred for 2 h before being centrifuged at 13,400 rpm for 15 min, at 4 °C. In this case, three washes with water were carried out to remove the ionic liquid. Lyophilization was carried out under the experimental conditions described in Section 2.3. 

The characteristic parameters (DLC and EE) of the Curc-SFNs 2 obtained were also indirectly calculated by the above expressions from the measurements of UV-Vis absorbance of curcumin.

### 2.5. Curcumin Release from Curc-SFNs

Curcumin release studies were carried out with Curc-SFNs 1 and Curc-SFNs 2. Twenty milligrams of Curc-SFNs were dispersed in 1 mL of PBS 1× (0.5% Tween 80) by ultrasonication. The samples were incubated at 37 °C in a tube rotator for 3 days to study curcumin release. At predetermined time points (0.5, 1, 1.5, 2, 3, 3.5, 4, 5, 25, 50 and 73 h), the samples were centrifuged at 13,400 rpm and the curcumin in the supernatant was monitored using UV-Vis spectrophotometry at 421 nm. The PBS solution was replaced with the same volume (1 mL) of fresh solution. The release experiments were carried out in triplicate for every sample. Experimental data were fitted using four release kinetic models found in the literature (zero order, first order, Higuchi and Ritger–Peppas) [67] to know the release mechanism of curcumin from the Curc-SFNs in PBS.

### 2.6. Characterization of SFNs and Curc-SFNs

#### 2.6.1. Dynamic Light Scattering (DLS)

The mean hydrodynamic diameter (Z-average), the Polidispersity Index (PdI), the Zeta Potential and the Electrophoretic Mobility were measured using a Zetasizer Nano ZSP instrument (Malvern Instruments Ltd., Worcestershire, UK) by DLS. All measurements were performed in purified water at 25 °C, a 173° angle relative to the source and with a nanoparticle concentration of 0.66 mg/mL. The influence of the SFNs concentration on size distribution and Zeta Potential was evaluated and it was checked that both parameters were practically constant in the range from 0.30 to 40 mg/mL and they remained constant up to 3 months. The mean values of the measurements performed in triplicate are reported.

#### 2.6.2. Field Emission Scanning Electron Microscopy (FESEM)

The morphology of the nanoparticles was examined by FESEM using an FEI Scios^TM^ microscope (Thermo Scientific, Waltham, MA, USA). The sample was placed on a pedestal as powder and then coated with gold.

#### 2.6.3. Transmission Electron Microscopy (TEM)

Sample preparation for TEM was carried out using 0.06 mg/mL nanoparticle suspensions in purified water and ultrasonification for 3 min with 30% amplitude. A drop of this suspension was placed on a 200 mesh copper grid coated with carbon. Once the drop was dried at room temperature, a drop of uranyl acetate was added and the copper grid was imaged in an FEI Tecnai^TM^ 12 microscope (Thermo Scientific, Waltham, MA, USA) operated at an acceleration voltage of 120 kV.

#### 2.6.4. Attenuated Total Reflectance Fourier Transformed Infrared Spectroscopy (ATR-FTIR) 

ATR-FTIR analysis was performed to detect the possible structural changes of SF after loading with curcumin. Each spectrum was acquired on a Nicolet^TM^ iS5 spectrometer (Thermo Scientific, Waltham, MA, USA), equipped with an iD3 ATR accessory (Thermo Scientific, Waltham, MA, USA) controlled by OMNIC Software Ver. 6.1.0.0038 (Waltham, MA, USA), measuring in absorbance mode with a resolution of 4 cm^−1^, a spectral range of 4000–550 cm^−1^, and 64 scans, using N-B strong apodization and mertz phase correction. 

#### 2.6.5. UV-Vis Absorbance Spectroscopy

Absorbance spectroscopy was performed on a Synergy MX UV-Vis spectrometer (BioTek Instruments Inc., Winooski, VT, USA). Samples were scanned from 230 to 700 nm in 96 well non-sterile acrylic Costar UV Plates, (N° 3635) (Corning, NY, USA), with UV transparent flat bottom.

#### 2.6.6. Fluorescence Spectroscopy

Fluorescence was studied on a Synergy MX UV-Vis spectrometer (Bio Tek Instruments Inc., Winooski, VT, USA). The fluorescence excitation spectra of curcumin samples or Curc-SFNs were obtained using 96 well non-sterile Costar black stripwell plates (N° 3914) (Corning, NY, USA) by varying excitation from 300 to 510 nm (5 nm step) and recording emission intensity at 530 nm. Fluorescence emission spectra of curcumin samples or Curc-SFNs were obtained by exciting at 420 nm and monitoring emission from 450 to 700 nm (5 nm steps). 

### 2.7. Free Radical Scavenging Activity of Curc-SFNs (DPPH Assay)

It is well known that substances which have radical scavenging activity such as curcumin induce color bleaching of a 2,2-diphenyl-1-picrylhydrazyl radical (DPPH·) solution, which can be measured spectrophotometrically [14]. To assess the DPPH· free radical scavenging capacity of the samples, the method of Blois [68] was used with slight modifications. Briefly, aliquots of 100 μL of the 1 mM (100 nmol) DPPH· stock solution in methanol (prepared daily and protected from light) were added to the Eppendorf^®^ vials containing a mixture of 800 μL of methanol and 100 μL of a water suspension of the samples at 1 mg/mL of curcumin or Curc-SFNs. 

Vials were thoroughly mixed and kept in darkness for 30 min. In the same conditions, ascorbic acid solutions (0–300 nmol in methanol) were tested as positive controls. After 30 min of reaction at 25 °C in darkness, the suspensions were centrifuged in an Eppendorf Centrifuge 5415D (20 min, 16,100 *g* at room temperature) to remove particles in the supernatants that could affect the spectrophotometric measurements. The radical scavenging was evaluated by measuring the absorbance at 515 nm, by using a Synergy MX UV-Vis spectrometer (BioTek Instruments Inc., Winooski, VT, USA) adding 200 μL/well. The radical scavenging activity was presented as Ascorbic Acid Equivalents per milligram of Curc-SFNs (nmol AAE/mg Curc-SFNs). All tests were done in quintuplicate and results were expressed as mean ± standard deviation (SD).

### 2.8. In Vitro Cytotoxicity Studies

Three cell lines were studied: human hepatocellular carcinoma (Hep3B), human neuroblastoma (Kelly Cells) and human bone marrow-derived mesenchymal stem cells (hBMSCs). Hep3B cells were obtained from the American Tissue Culture Collection (ATCC, Manassas, VA, USA) and Kelly Cells were purchased from Sigma Aldrich (St. Louis, MO, USA). The hBMSCs were obtained as previously described in a paper with silk materials and curcumin of the Kaplan research group [44]. The culture media used were Roswell Park Memorial Institute (RPMI) for Hep3B and Kelly Cells and Dulbecco’s modified Eagle’s medium (DMEM) for hBMSCs. Both media were supplemented with 10% fetal bovine serum, 1% penicillin and streptomycin, 1% non-essential amino acids and 1 ng/mL basic fibroblast growth factor (human recombinant, Invitrogen), at 37 °C in a 5%-CO_2_ incubator. Cells were culturally expanded using trypsin passaging at a 1:3 split ratio. The medium was changed twice a week. The size distribution was stable in cell culture media DMEM with 10% fetal bovine serum up to 72 h.

For cytotoxicity studies, the three cell lines were seeded onto 96-well tissue culture plates at a density of 1.0 × 10^4^ cells/well (for Hep3B and hBMSCs) and 2.0 × 10^4^ cells/well (for Kelly Cells) and left for 24 h, after which the growth medium was removed and replaced with the medium containing SFNs, Curc-SFNs 1 or Curc-SFNs 2. In each experiment, growth medium without nanoparticles was used as a control. After 24 h, the culture medium of each well was replaced with fresh medium containing SFNs or Curc-SFNs in a wide concentration range: from 1.62 to 6650 μg/mL. Initial suspensions of nanoparticles in ultrapure water were previously sonicated 3 min with 30% amplitude and then diluted to 10% (*v*/*v*) with the medium. After 48 h, the medium was removed and AlamarBlue^®^ assay (ThermoFisher Scientific, Waltham, MA, USA) was performed following the manufacturer’s protocol. Then, post reaction medium aliquots (90 μL) were transferred to black 96-well plates and quantified for fluorescence intensity within a SpectraMax M2 plate reader (Molecular Devices, Sunnyvale, CA, USA) using an excitation wavelength of 560 nm and an emission wavelength of 590 nm. The data are presented as mean values ± SD calculated from at least three samples per condition.

## 3. Results and Discussion

### 3.1. Characterization of SFNs and Curc-SFNs

#### 3.1.1. Dynamic Light Scattering (DLS)

**Figure 1:**
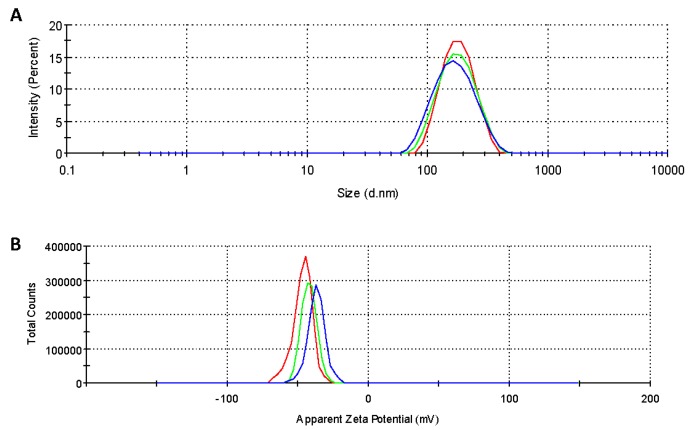
Characterization of silk fibroin nanoparticles (blue), curcumin-loaded silk fibroin nanoparticles synthesized by physical adsorption (green) and curcumin-loaded silk fibroin nanoparticles synthesized by coprecipitation (red): (**A**) size distribution; and (**B**) Zeta Potential measured at 25 °C in purified water.

The SFNs, Curc-SFNs 1 and Curc-SFNs 2 were characterized by DLS to ascertain their hydrodynamic diameter (expressed as Z-average), PdI, Zeta Potential and Electrophoretic Mobility (Table 1). Figure 1 shows the size and the Zeta Potential distributions of the SFNs and the Curc-SFNs obtained by both methods. 

The results showed that SFNs had a lower diameter while the PdI was similar (and lower than 0.15) for all of the samples, resulting in near monodisperse size distributions. The Zeta Potential of the SFNs and Curc-SFNs was in the same range and sufficiently negative, which is especially relevant if nanoparticles are to have high colloidal stability. The absolute Zeta Potential values of the SFNs was higher than the values found in the literature for SFNs obtained by other classical methods [50,54,69,70], which reflects the improvement in stability of the SFNs and hence of the Curc-SFNs obtained with this new procedure. 

#### 3.1.2. Transmission Electron Microscopy (TEM)

**Figure 2:**
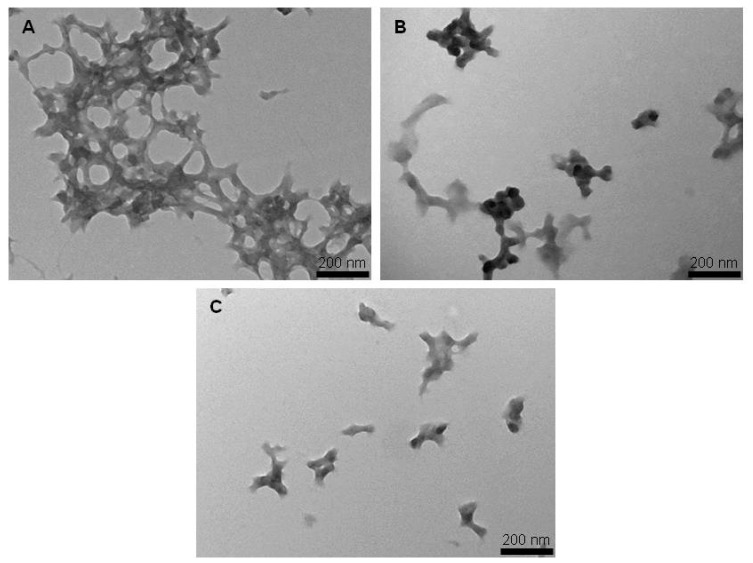
Transmission Electron Microscopy images of: (**A**) silk fibroin nanoparticles; (**B**) curcumin-loaded silk fibroin nanoparticles synthesized by physical adsorption; and (**C**) curcumin-loaded silk fibroin nanoparticles synthesized by coprecipitation (59,000×).

To detect any differences in the size and morphology of SFNs, Curc-SFNs 1 and Curc-SFNs 2, TEM was performed. The TEM observations (Figure 2 and Figure S2) showed globular granules for SFNs and Curc-SFNs 1 with some aggregation and a slightly elongated shape for Curc-SFNs 2. The granules were about 60–95 nm in diameter. This diameter range was obtained from analysis of the TEM images using GIMP software (ver. 2.8, Mountain View, CA, USA). Unlike the more defined shape of the Curc-SFNs 1, the SFNs and Curc-SFNs 2 were uneven in appearance.

#### 3.1.3. Field Emission Scanning Electron Microscopy (FESEM)

**Figure 3:**
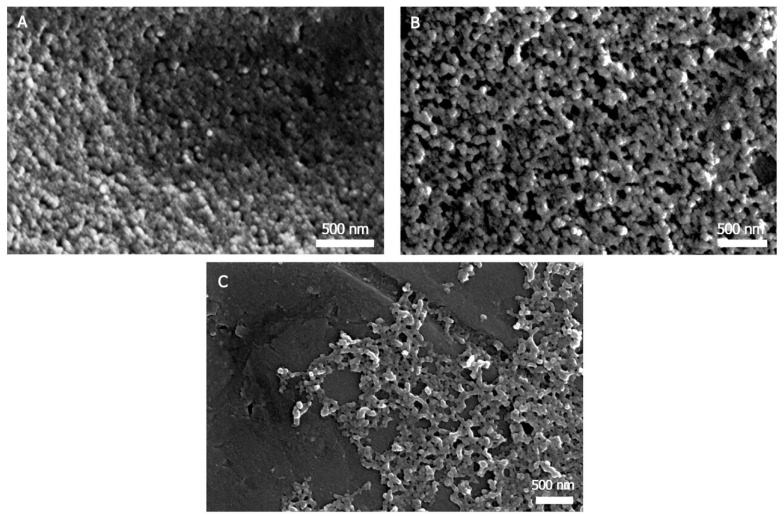
Field Emission Scanning Electron Microscopy pictures of: (**A**) silk fibroin nanoparticles; (**B**) curcumin-loaded silk fibroin nanoparticles synthesized by physical adsorption; and (**C**) curcumin-loaded silk fibroin nanoparticles synthesized by coprecipitation.

FESEM is a commonly used technique to study the morphology and size of a variety of nanoparticles. FESEM observation (Figure 3) of these nanoparticles provided similar results to the TEM analysis. Figure 3 shows nanospherical morphology for SFNs and Curc-SFNs 1 and again a more elongated shape for Curc-SFNs 2. The TEM and FESEM images revealed smaller diameters compared with the DLS results, probably due to swelling of the particles in the water solution, as the DLS measurements were performed with suspensions of the particles in water, while the TEM and FESEM samples were dried. This difference has also been observed by other authors [53,69]. 

#### 3.1.4. Attenuated Total Reflectance Fourier Transformed Infrared Spectroscopy

Information about the secondary structure of the proteins can be obtained from the position of the infrared signals and their relative intensity, as well as identifying specific molecules by comparison of their spectra with the databases [69]. In the present study, we used spectroscopy to verify that nanoparticles retained the β-sheet structure after the curcumin loading process. The Curc-SFNs spectra were coincident with the spectrum of nanoparticles prepared by the method described by Zhang et al. [71] by dissolving SF in the Ajisawa solvent system [65] (Figure S3). The spectrum of the Curc-SFNs showed the characteristic β-sheet signals, such as 1624–1626 cm^−1^ (Amide I, C=O Stretching), 1517–1521 cm^−1^ (Amide II, N–H Bending) and 1232 cm^−1^ (Amide III, C–H Stretching), as in the literature [69,71,72], confirming that β-sheet in the SFNs was not modified after the curcumin loading processes, either by coprecipitation or by adsorption. The main signals in the spectrum of curcumin were 1627, 1603, 1507, 1428, 1283, 1233, 1205, 1154, and 1114 cm^−1^, which match those previously described in the literature [73] (Figure S3). In the Curc-SFNs spectra, the intense absorbance of the SF signals, in addition to a much larger mass ratio, masked the curcumin signals.

#### 3.1.5. UV-Vis Absorbance Spectroscopy

Curcumin is a tautomeric compound that absorbs light in the visible range and displays a yellow color when dissolved in organic solvents such as chloroform, dimethylsulfoxide, ethanol, methanol or acetone. Curcumin is practically insoluble in aqueous media at neutral pH [74]. The UV-Vis spectrum of Curc-SFNs dispersed in ultrapure water had a peak with a λ_max_ = 435 nm, similar to free curcumin in low polar solvents such as octanol (λ_max_ = 430 nm). Curcumin dissolved in water had a poorly resolved wide band with λ_max_~450 nm [37,74,75] (Figure S4). As the photochemical properties of curcumin depend strongly on the microenvironment [76], this hypsochromic shift of the maximum absorption peak confirmed that the curcumin was in a more hydrophobic environment, or less exposed to the solvent, than in the free state.

#### 3.1.6. Fluorescence Spectroscopy

While significant curcumin fluorescence was not recorded in water, the nanoparticles loaded both by adsorption or by coprecipitation had similar fluorescence spectra, with a single peak for both excitation and emission Figure S5). This peak was similar to those found in the literature [33]. The maximum of the excitation spectrum appeared at 450 nm in both cases, although Curc-SFNs 1 had higher fluorescence intensity at the same particle concentration. The maximum emission spectrum of the nanoparticles was measured at 535 nm in both cases, the spectrum of Curc-SFNs 1 showed greater intensity. Both facts agree with the higher DLC of the nanoparticles. Figure S6b shows, from left to right, the fluorescence at 365 nm of free curcumin, Curc-SFNs 1 and Curc-SFNs 2 and SFNs at three different concentrations of nanoparticles: 10, 1 and 0.1 mg/mL. The only fluorescent samples were the Curc-SFNs obtained in this work. The different intensity of the fluorescence between the three cuvettes of each sample was due to the different concentration of the nanoparticle suspensions in water. Figure S6a shows the same samples but imaged with white light.

### 3.2. Drug Loading

Drug loading in three independent coprecipitation and physical adsorption experiments gave the results shown in Table 2. The DLC in the physical adsorption experiments was higher than in the coprecipitation assays, mainly because the initial curcumin/SF ratio (weight) was much lower in the latter. However, the EE had approximately the same value of around 50%. Both parameters (DLC and EE) were generally similar or higher than those obtained in similar studies using SFNs as drug vehicles [53,54]. To the best of our knowledge, only a recent study of Perteghella et al. [77] reached DLC values higher than 30% for Curc-SFNs. In this case, they were synthesized by acetone desolvation method. Differences in DLC values could be due to the synthesis technique. 

### 3.3. Curcumin Release from Curc-SFNs

**Figure 4:**
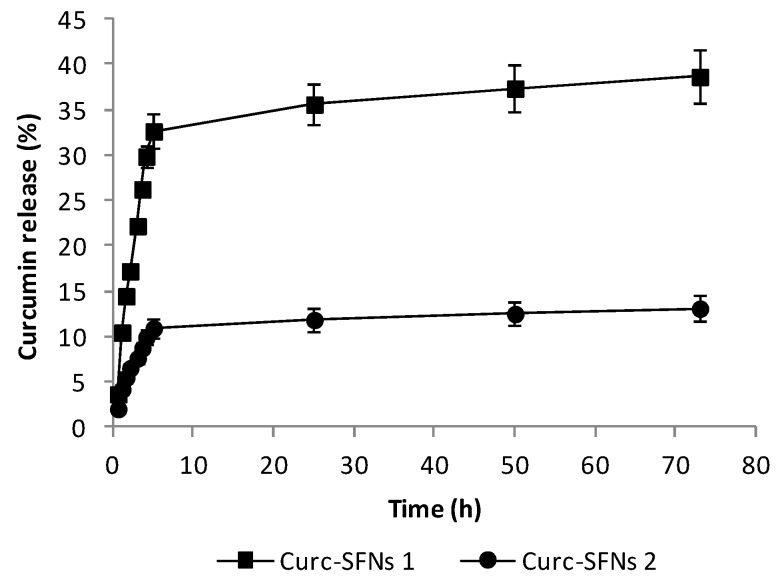
Curcumin release from curcumin-loaded silk fibroin nanoparticles synthesized by physical adsorption (Curc-SFNs 1) and curcumin-loaded silk fibroin nanoparticles synthesized by coprecipitation (Curc-SFNs 2) in PBS 1× (0.5% Tween 80) at 37 °C.

The release behavior of the two types of Curc-SFNs (Curc-SFNs 1 and Curc-SFNs 2) was evaluated. The studies were carried out at 37 °C in PBS 1× (with 0.5% Tween 80 due to the poor solubility of curcumin in PBS). A notable difference in the release rate was seen between the two Curc-SFNs. For both types of nanoparticles (Figure 4), burst release was observed in the first 5 h, becoming plateau after 24 h. Curcumin release was higher for the Curc-SFNs 1, reaching a maximum value of approximately 35% of the loaded drug, while in the case of the Curc-SFNs 2, the maximum value was around 10% (Figure 4). This difference in the release of the two Curc-SFNs was probably due to the lower drug loading of the Curc-SFNs 2. In addition, in the authors’ opinion, it might also be because Curc-SFNs 2, which are synthesized by coprecipitation, probably have the drug encapsulated inside the nanoparticle, making more difficult its release. On the contrary, in the case of Curc-SFNs 1, the drug is mainly adsorbed onto the surface so the release could be easier.

Curcumin release profiles (from 0.5 to 5 h) have been fitted to several release models: zero order, first order, Higuchi and Ritger–Peppas. Table 3 shows the fitting equations obtained and the goodness of the fittings. We found that curcumin release profiles of Curc-SFNs 1 and Curc-SFNs 2 can be better agreed with Higuchi equation, although all fittings are quite good for both types of Curc-SFNs. Higuchi equation is based on Fick’s Law where the release occurs by the diffusion of drugs within the delivery system [78]. This equation is a conceptually simple model but it is valid only under specific assumptions. It is known that, under some experimental conditions (swelling, glassy/rubbery transitions, dissolution or concentration-dependent diffusion), the release mechanism can deviate from Fickian diffusion following an Anomalous transport (non-Fickian) [79]. Therefore, when the drug release mechanism is not well known or more than one type of release phenomena is involved, a more generic equation is necessary [78]. In our case, to know the release mechanisms of curcumin from Curc-SFNs, Ritger–Peppas equation [80] has been used because of swellable silk fibroin matrix in PBS. Ritger and Peppas claimed that the mechanistic limits of the diffusional exponent, *n*, are dependent on the geometry (film, cylinder, and sphere) of the associated release device (Table 4) [80]. For Curc-SFNs 1 and Curc-SFNs 2, *n*, which is indicative of the transport mechanism, has a value of 0.80 ± 0.05 and 0.66 ± 0.04, respectively, indicating that the curcumin release from Curc-SFNs (assuming spheres) is controlled by Anomalous (non-Fickian) transport in both cases. This means that the curcumin release from Curc-SFNs is not a pure diffusional (Fickian) mechanism but involves a relaxational or convection mechanism which is usually associated with a major state or phase change [79]. The same drug release mechanism was found in the literature for polymeric microspheres loaded with simvastatin with DLC values similar to those presented in this work [67] and for polymeric nanoparticles containing diazepam [81].

### 3.4. Free Radical Scavenging Activity of Curcumin Loaded Nanoparticles (DPPH Assay)

The DPPH· assay has been previously used to test the free radical scavenging capacity of curcumin and curcumin-loaded nanocarriers [14,82]. In its radical form, DPPH· strongly absorbs at 515 nm, but its absorption decreases after reaction with an antioxidant. The radical scavenging activity of both free curcumin and curcumin-loaded nanoparticles Curc-SFNs (Curc-SFNs 1 and Curc-SFNs 2) have been evaluated and compared to the equivalents of an antioxidant standard needed to obtain the same absorption reduction in the same reaction conditions. It should be noted that two equivalents of ascorbic acid react with one equivalent of DPPH· but Curcumin interacts in the ratio 1:1 [70]. Thus, the Ascorbic Acid Equivalents per milligram of loaded nanoparticles in aqueous solutions obtained for the curcumin loaded nanoparticles varied from 8.15 ± 1.17 nmol AAE/mg Curc-SFNs 1 to 4.93 ± 0.92 nmol AAE/mg Curc-SFNs 2, while the empty nanoparticles also exert a small effect (1.1 ± 0.1 nmol AA/mg SFNs). Silk fibroin contain about a 4% of aromatic residues that present radical scavenging activity as previously reported [59,83].

These results agree with the DLC of curcumin in nanoparticles, previously described in Drug Loading Section, and remarks that curcumin retain the full radical scavenging activity after being adsorbed onto SFNs in the assay conditions. This antioxidant protective effect of SFNs has been previously described with other sensitive phenolic compounds as Resveratrol [52] or Quercetin [59].

### 3.5. In Vitro Cytotoxicity Studies

**Figure 5:**
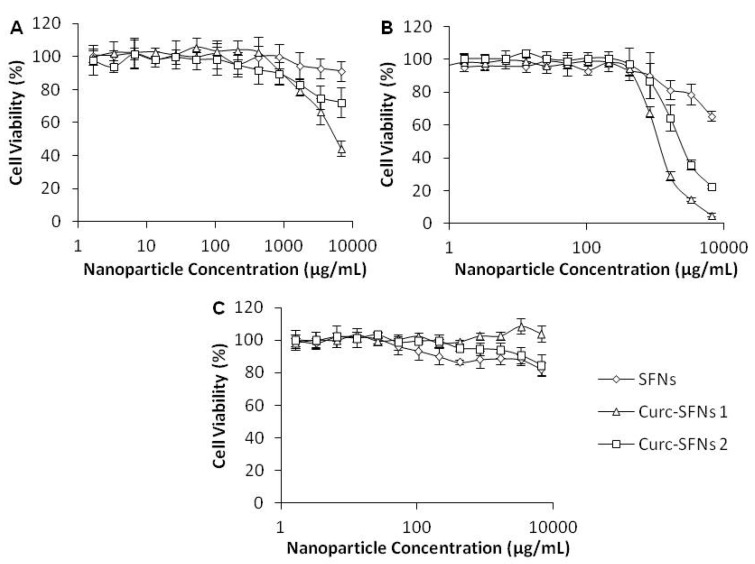
In vitro cytotoxicity studies after 48 h of exposure to silk fibroin nanoparticles (SFNs), curcumin-loaded silk fibroin nanoparticles synthesized by physical adsorption (Curc-SFNs 1) and curcumin-loaded silk fibroin nanoparticles synthesized by coprecipitation (Curc-SFNs 2): (**A**) Hep3B cells; (**B**) Kelly cells; and (**C**) hMBSCs.

The cytotoxicity of SFNs, Curc-SFNs 1 and Curc-SFNs 2 were evaluated after 48 h of exposure using AlamarBlue^®^ in three cell lines: Hep3B cells, Kelly cells and hBMSCs. We found similar trends for the tumor cell lines (Hep3B cells and Kelly cells) with regard to nanoparticle cytotoxicity (Figure 5a,b). After 48 h of incubation with several concentrations of nanoparticles, the results showed that cell viability slowly decreased with an increased concentration of nanoparticles. In addition, Curc-SFNs were more cytotoxic than SFNs, while the Curc-SFNs 1 were more cytotoxic than Curc-SFNs 2. These findings suggest that nanoparticle cytotoxicity is dependent on drug concentration because the Curc-SFNs 1 had a higher DLC than Curc-SFNs 2 (Table 2). However, in the case of healthy cells (hBMSCs), no cytotoxicity was observed, even after exposure to the most heavily drug loaded-SFNs (Figure 5c). Similar results were obtained by Chang et al. [84], who used spherical amphiphilic nanoparticles as a curcumin carrier. These authors found that the curcumin loaded-amphiphilic nanoparticles had significant selective cytotoxicity against MG-63 osteosarcoma cells compared with normal osteoblasts. The fact that curcumin was non-toxic to healthy cells has been shown in several previous works [85,86], which established the potential of curcumin as a chemopreventive and chemotherapeutic agent in malignancy. The Curc-SFNs obtained by the two different procedures were cytotoxic for the three cell lines in the order: Kelly cells > Hep3B cells > hBMSCs.

In cancer cells, metabolic pathways are reprogrammed to satisfy tumor cell proliferation and survival requirements. In these cells, glycolysis and glutaminolysis are strongly increased. These metabolic processes and the role of mitochondria in supporting tumor cell metabolism are probably the reason of the different behavior between Hep3B, Kelly Cells and hBMSCs. Syng-ai et al. [86] showed that curcumin induce apoptosis in human breast carcinoma cell lines as well as in human hepatoma cells but failed to do so in normal rat hepatocyte primary cultures. Their results indicate that Glutathione (GSH, also known as γ-l-glutamyl-l-cysteineglycine) plays a vital role in the sensitivity of these cell lines to curcumin. Depletion of GSH further sensitized the cells to curcumin effects, and the cell death is caused by the generation of reactive oxygen species. Curcumin also down-regulated the expression of bcl-2 protein in tumor cells, which may be responsible for making them vulnerable to apoptotic death.

## 4. Conclusions

Curc-SFNs were successfully synthesized by two environmentally friendly procedures using ILs and high-power ultrasound to dissolve the SF. High DLC and EE values were obtained in both cases compared with those in the literature, and curcumin release was influenced by the synthesis method of the Curc-SFNs. The SFNs and the Curc-SFNs obtained showed a narrow size distribution, with a hydrodynamic diameter of <175 nm, and high Zeta Potential (in absolute terms), which make them excellent nanocarriers for use in therapeutic treatments. The nanospherical morphology was confirmed by FESEM and TEM, and the curcumin was fluorescent when encapsulated or adsorbed on the SFNs but not in its free state. The antioxidant activity against DPPH· showed that the curcumin loaded in Curc-SFNs retains full antioxidant activity. The Curc-SFNs obtained in this paper enhanced the antitumor activity of curcumin towards the two different tumor cell lines studied, while the viability of the healthy cells did not decrease. This work broadens the possibility of using these SFNs, which have been synthesized by an industrial process, as future systems for other drugs of hydrophilic or hydrophobic nature, such as curcumin.

## Figures and Tables

**Table 1:** Physical characterization of the silk fibroin nanoparticles (SFNs), curcumin-loaded silk fibroin nanoparticles synthesized by physical adsorption (Curc-SFNs 1) and curcumin-loaded silk fibroin nanoparticles synthesized by coprecipitation (Curc-SFNs 2).

Sample	Z-Average (nm) ^1^	PdI ^1^	Zeta Potential (mV) ^1^	Electrophoretic Mobility (μm·cm/Vs) ^1^
SFNs	157.9 ± 1.5	0.132 ± 0.011	−41.3 ± 0.6	−3.396 ± 0.146
Curc-SFNs 1	166.0 ± 0.1	0.114 ± 0.003	−42.9 ± 2.8	−3.362 ± 0.264
Curc-SFNs 2	171.2 ± 2.6	0.106 ± 0.017	−45.9 ± 5.0	−3.504 ± 0.348

^1^ Mean Values ± SD (standard deviation).

**Table 2:** Drug loading and encapsulation efficiency of the curcumin-loaded silk fibroin nanoparticles synthesized by physical adsorption (Curc-SFNs 1) and curcumin-loaded silk fibroin nanoparticles synthesized by coprecipitation (Curc-SFNs 2).

Parameter	Curc-SFNs 1 ^1^	Curc-SFNs 2 ^1^
DLC (%)	6.63 ± 0.09	2.47 ± 0.11
EE (%)	53.75 ± 0.81	48.84 ± 2.67

^1^ Mean Values ± SD (standard deviation) (*n* = 3).

**Table 3:** Release models of curcumin-loaded silk fibroin nanoparticles synthesized by physical adsorption (Curc-SFNs 1) and curcumin-loaded silk fibroin nanoparticles synthesized by coprecipitation (Curc-SFNs 2).

Release Model	Curc-SFNs 1	Curc-SFNs 2
Zero order	*y* = 6.36*x* + 2.92	*y* = 1.89*x* + 1.98
	*R*^2^ = 0.9738	*R*^2^ = 0.9661
First order	*y* = 56.89(1 × 10^−0.17*x*^)	*y* = 13.61(1 × 10^−0.31*x*^)
	*R*^2^ = 0.9909	*R*^2^ = 0.9872
Higuchi	*y* = 19.51*x*^0.5^ − 10.60	*y* = 5.83*x*^0.5^ − 2.07
	*R*^2^ = 0.9948	*R*^2^ = 0.9916
Ritger–Peppas	*y* = 9.41*x^n^*	*y* = 3.85*x^n^*
	*R*^2^ = 0.9856	*R*^2^ = 0.9866
	*n* = 0.80 ± 0.05	*n* = 0.66 ± 0.04

**Table 4:** Diffusional exponent *n* of the Ritger–Peppas equation and drug release mechanism from polymeric controlled delivery system for different geometries [82].

Thin Film	Cylinder	Sphere	Drug Release Mechanism
*n* = 0.5	*n* = 0.45	*n* = 0.43	Fickian diffusion
0.5 < *n* < 1	0.45 < *n* < 0.89	0.43 < *n* < 0.85	Anomalous (non-Fickian) transport
*n* = 1.0	*n* = 0.89	*n* = 0.85	Case-II transport

## Supplementary Materials

The following are available online at http://www.mdpi.com/2079-4991/8/2/126/s1, Figure S1: Chemical structure of curcumin, Figure S2: Extra TEM images of: (a) SFNs; (b) Curc-SFNs 1; and (c) Curc-SFNs 2, Figure S3: (Left) A comparative ATR-FTIR full spectrum of: (a) curcumin; (b) Curc-SFNs 1; (c) Curc-SFNs 2; and (d) SFNs as negative control. (Right) The 1700-1100 cm^−1^ region of the same spectra, Figure S4. UV/Vis absorbance corrected spectra in ultrapure water (after subtracting the SFN spectrum) of: curcumin (red), Curc-SFNs 1 (pink), Curc-SFNs 2 (blue) and the SFNs spectrum (black), Figure S5: Fluorescence excitation (solid) and emission (dotted) spectra of Curc-SFNs 1 (pink) and Curc-SFNs 2 (blue), Figure S6. Comparison of suspensions of SFNs, free curcumin and Curc-SFNs. From left to right: free curcumin, Curc-SFNs 1, Curc-SFNs 2 and SFNs. The three cuvettes for each sample represent three different concentrations of the suspensions of nanoparticles in water; from left to right: 10 mg/mL, 1 mg/mL and 0.1 mg/mL. Figure S6a is with white light and Figure S6b at 365 nm.
