# Supplementary material for: Production of Curcumin-Loaded Silk Fibroin Nanoparticles for Cancer Therapy
Source: Nanomaterials (Basel). 2018 Feb 24;8(2):126. doi: 10.3390/nano8020126 (PMC5853757; doi:10.3390/nano8020126)
Supplement: Supplementary file 1 [file nanomaterials-08-00126-s001.pdf]

# Supplementary materials

# Production of Curcumin-Loaded Silk Fibroin Nanoparticles for Cancer Therapy

**Mercedes G. Montalbán [1,*], Jeannine M. Coburn [2,3], A. Abel Lozano-Pérez [4], José L. Cenis [4], Gloria Víllora [1] and David L. Kaplan [2]**

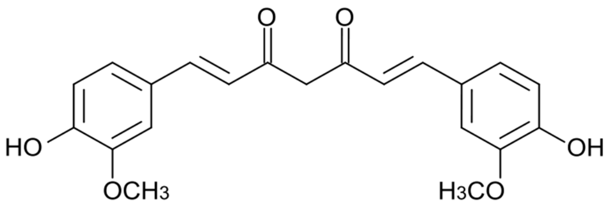


**Figure S1.** Chemical structure of curcumin.

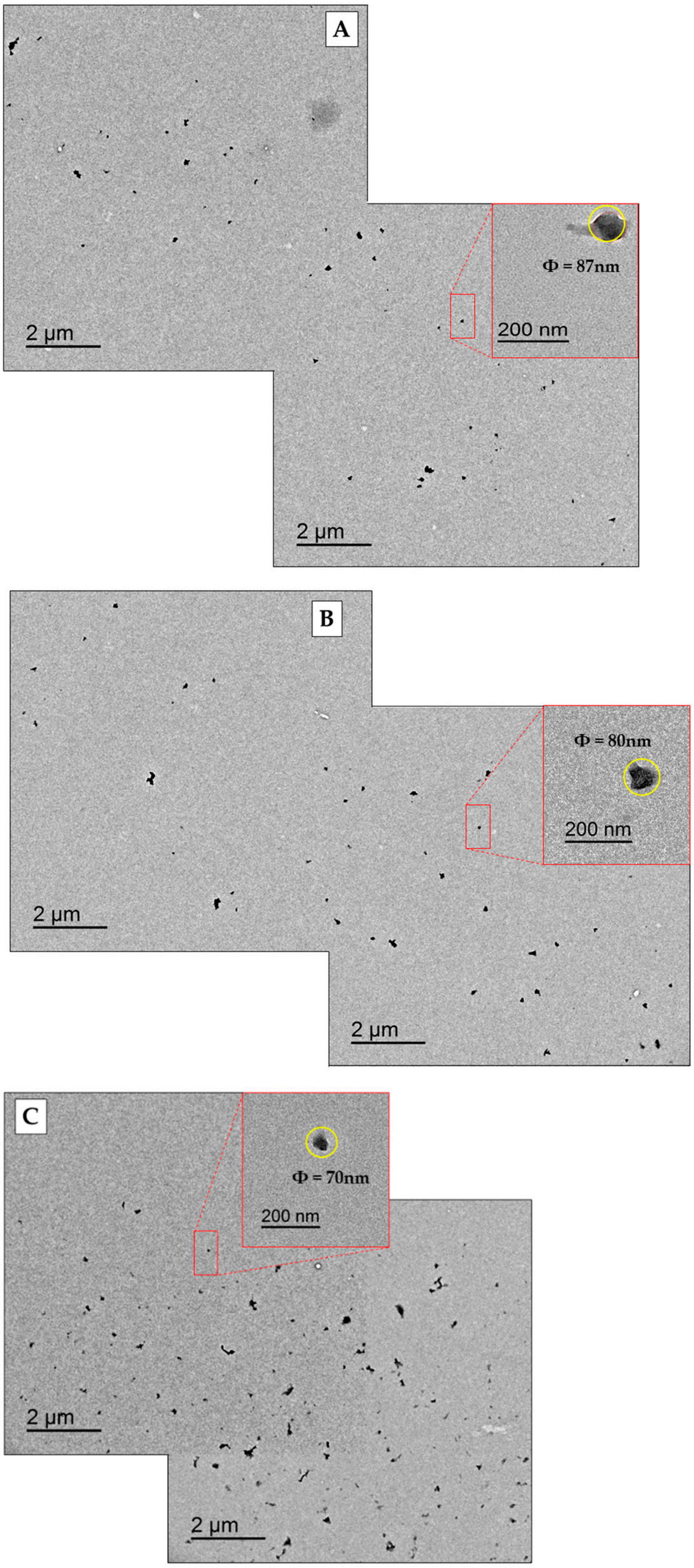


**Figure S2.** Extra TEM images of (**a**) SFNs; (**b**) Curc-SFNs 1 and (**c**) Curc-SFNs 2.

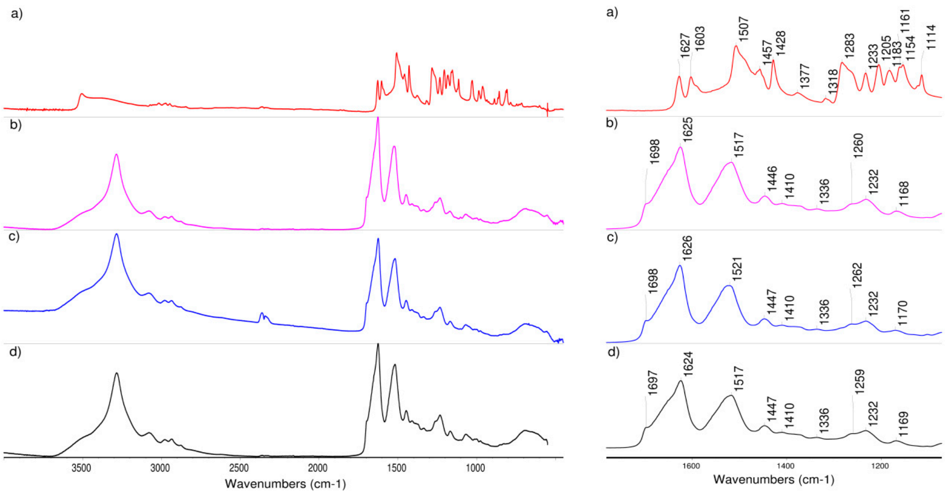


**Figure S3.** On the left, a comparative ATR-FTIR full spectrum of (a) curcumin; (b) Curc-SFNs 1; (c) Curc-SFNs 2; (d) SFNs as negative control. On the right, the 1700–1100 $cm^{-1}$ region of the same spectra.

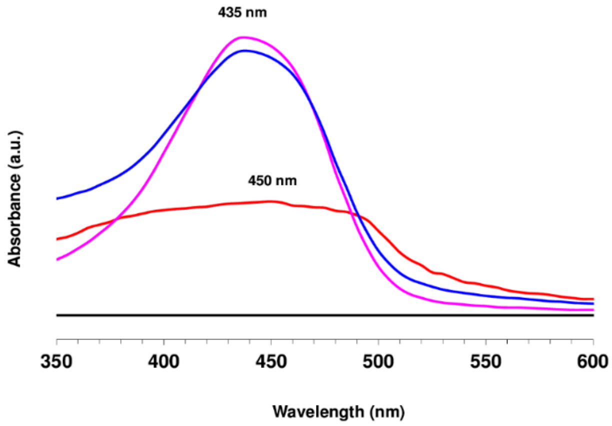


**Figure S4.** UV/Vis absorbance corrected spectra in ultrapure water (after subtracting the SFN spectrum) of: curcumin (red), Curc-SFNs 1 (pink), Curc-SFNs 2 (blue) and the SFNs spectrum (black).

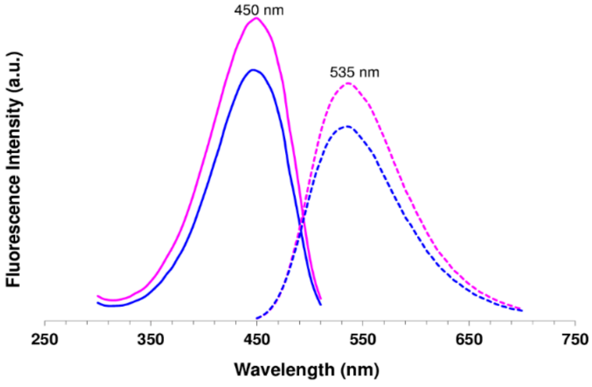


**Figure S5.** Fluorescence excitation (solid) and emission (dotted) spectra of Curc-SFNs 1 (pink) and Curc-SFNs 2 (blue).

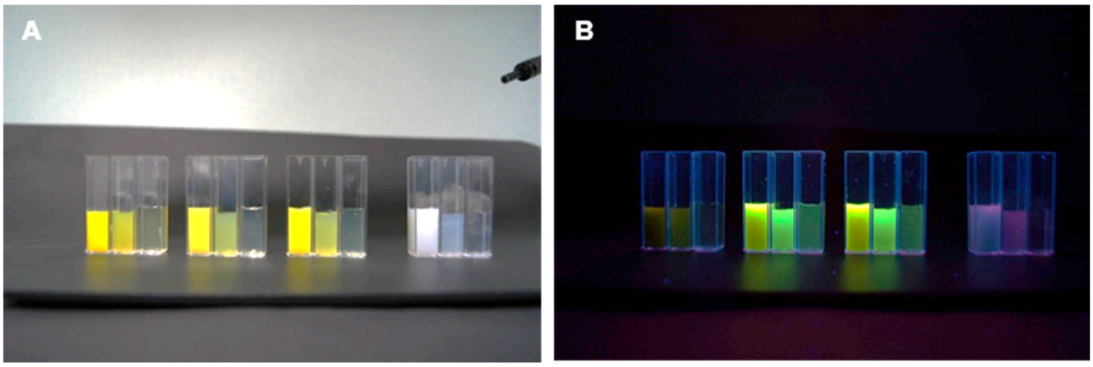


**Figure S6.** Comparison of suspensions of SFNs, free curcumin and Curc-SFNs. From left to right: free curcumin, Curc-SFNs 1, Curc-SFNs 2 and SFNs. The three cuvettes for each sample represent three different concentrations of the suspensions of nanoparticles in water; from left to right: 10 mg/mL, 1 mg/mL and 0.1 mg/mL. **Figure S6A** is with white light and **Figure S6B** at 365 nm.
